# A comparison of pulsed radiofrequency and radiofrequency denervation for lumbar facet joint pain

**DOI:** 10.1186/s13018-023-03814-5

**Published:** 2023-05-05

**Authors:** Shao-Jun Li, Shu-Li Zhang, Dan Feng

**Affiliations:** Department of Pain Management, Wuhan No.1 Hospital, Wuhan, 430022 Hubei Province China

**Keywords:** Pulsed radiofrequency, Radiofrequency denervation, Posterior branches, Lumbar facet joint pain

## Abstract

**Background:**

Lumbar facet joint pain is a common disorder. The main symptom is chronic lumbar pain, which can reduce quality of life. Radiofrequency has often been used to treat lumbar facet joint pain. However, the effectiveness of this technique has been controversial. This study was conducted to compare the effectiveness of pulsed radiofrequency (PRF) and radiofrequency denervation (RD) for lumbar facet joint pain.

**Methods:**

One hundred and forty-two patients with lumbar facet joint pain were allocated to two treatment groups: PRF group (*N* = 72) and RD group (*N* = 70). Patients enrolled in the study were assessed using a visual analogue scale (VAS), Roland-Morris questionnaire (RMQ), Oswestry disability index (ODI) and Short-Form 36 (SF-36) questionnaire before therapy, 3 months and 12 months later.

**Results:**

There were no significant differences in VAS, RMQ score, ODI score and SF-36 score at 3 months (*p* > 0.05). Significant differences in pain control were observed in both groups at 12 months (3.09 ± 1.72 vs. 2.37 ± 1.22, *p* = 0.006). There was a significant difference in RMQ score (11.58 ± 3.58 vs. 8.17 ± 2.34, *p* < 0.001) and ODI score (43.65 ± 11.01 vs. 35.42 ± 11.32, *p* < 0.001) at 12 months. The total SF-36 score was higher in the RD group than in the PRF group at 12 months (58.45 ± 6.97 vs. 69.36 ± 6.43, *p* < 0.001). In terms of complications, skin numbness occurred in three patients. Mild pain such as burning and pinking at the puncture site in two patients. One patient experienced a decrease in back muscle strength and back muscle fatigue. These complications disappeared in 3 weeks without any treatment. There were no serious adverse events in the PRF group.

**Conclusion:**

Radiofrequency is an effective and safe treatment option for patients with lumbar facet joint pain. RD could provide good and lasting pain relief, with significant improvement in lumbar function and quality of life at long-term follow-up.

## Introduction

Lumbar facet joint pain is a common disorder and has been attributed to substantial healthcare costs. Approximately 80% of people experience low back pain in their lifetime, and lumbar facet joint pain affects more women than men [1, 2]. Although persistent back pain can result from a variety of causes, including intervertebral disk, ligamentous, facet joint and nerve dysfunction, facet joint dysfunction is considered a critical factor in the development of chronic low back pain. The prevalence of lumbar facet joint pain varies widely, ranging from 15 to 45% [3–5]. Lumbar facet joint pain is a symptom characterized by pain in the spine and paraspinal region. Patients with lumbar facet joint pain usually do not have neurological symptoms. Acute lumbar facet joint pain is defined as lasting less than 1 month and is considered a self-limited condition that often does not require treatment. With time, recurrent pain persisting more than 3 months after onset is considered as chronic lumbar facet joint pain. Generally, an episode of acute low back pain does not seriously affect work and daily life, whereas persistent back pain, especially from the lumbar facet joints, can lead not only to significant activity limitations but also to increased disability rates and healthcare costs [1]. Therefore, controlling pain and improving quality of life are the main goals in the treatment of lumbar facet joint pain.

Radiofrequency is a minimally invasive technique for providing lasting pain relief to patients with lumbar facet joint pain. There are two types of radiofrequency therapy, pulsed radiofrequency (PRF) and radiofrequency denervation (RD). In RRF therapy, the radiofrequency generator produces alternating repetitive electrical stimulation to keep the temperature of the nerve tissue below 42 °C [6]. In addition, the short duration and rest period between pulses can prevent apoptosis and necrosis of histiocytes [7]. A previous study showed that PRF may selectively have a greater effect on the smaller pain-carrying fibers, such as c- and a-delta fibers, and little effect on the larger A-β neurons that mediate non-pain-related sensations [8, 9]. For RD therapy, a radiofrequency generator produces an alternating current, which can induce ionic movements in the tissue directly surrounding the needle tip [10]. Typically, the temperature of the electrode tip is 60–80 °C for 60–90 s, resulting in selective thermal coagulation of pain-carrying nerve fibers (A and C fibers) [11].

The effectiveness of radiofrequency has been well studied. Sansone et al. [12] reported that PRF was a promising technique in the management of lumbar facet joint pain. The procedure was well tolerated and reliable when nerve endings were regrown. Another study has found that PRF of the lumbar medial branches provided good pain relief for at least 6 months in 70% of patients [13]. Shealy et al. [14] were the first to report that conventional radiofrequency denervation can be an effective alternative in the treatment of facet joint pain. Afifi et al. [15] concluded that RD was associated with improved pain relief and quality of life compared with chemical neurolysis for facet joint-related chronic lower back pain. However, Tekin et al. [16] found that PRF had short-term effects on lumbar facet joint pain. Studies have found low evidence for RD treatment of lumbar facet joint pain [17, 18]. There is no consensus on the effectiveness of radiofrequency treatment for lumbar facet joint pain. The aim of this study was to evaluate the long-term outcomes comparing two different methods of radiofrequency: PRF treatment and RD treatment. The study focused on outcomes in terms of pain control, lumbar function and quality of life.

## Methods

This was a retrospective observational study and was conducted between October 2020 and December 2021 at the Department of Pain Management of Wuhan No.1 Hospital. In this study, 163 patients with lumbar facet joint pain were reviewed. The study was conducted in accordance with the principles of the Declaration of Helsinki and the guidelines of Wuhan No.1 Hospital. Informed consent was obtained from all individual participants included in the study. The follow-up period ended in February 2023.

Inclusion criteria were: low back pain persisting for ≥ 3 months in duration; no neurological symptoms; focal tenderness over one or more facet joints; persistent low back pain, hip pain without radicular syndrome; ineffective for conservative treatment such as physiotherapy, chiropractic manipulation, exercise and medical therapy.

Exclusion criteria were: neurological low back pain; age > 85 years or < 18 years; coagulopathy; infection including puncture site, lung or urinary tract; tumors, tuberculosis or severe lumbar kyphosis.

**Fig. 1:**
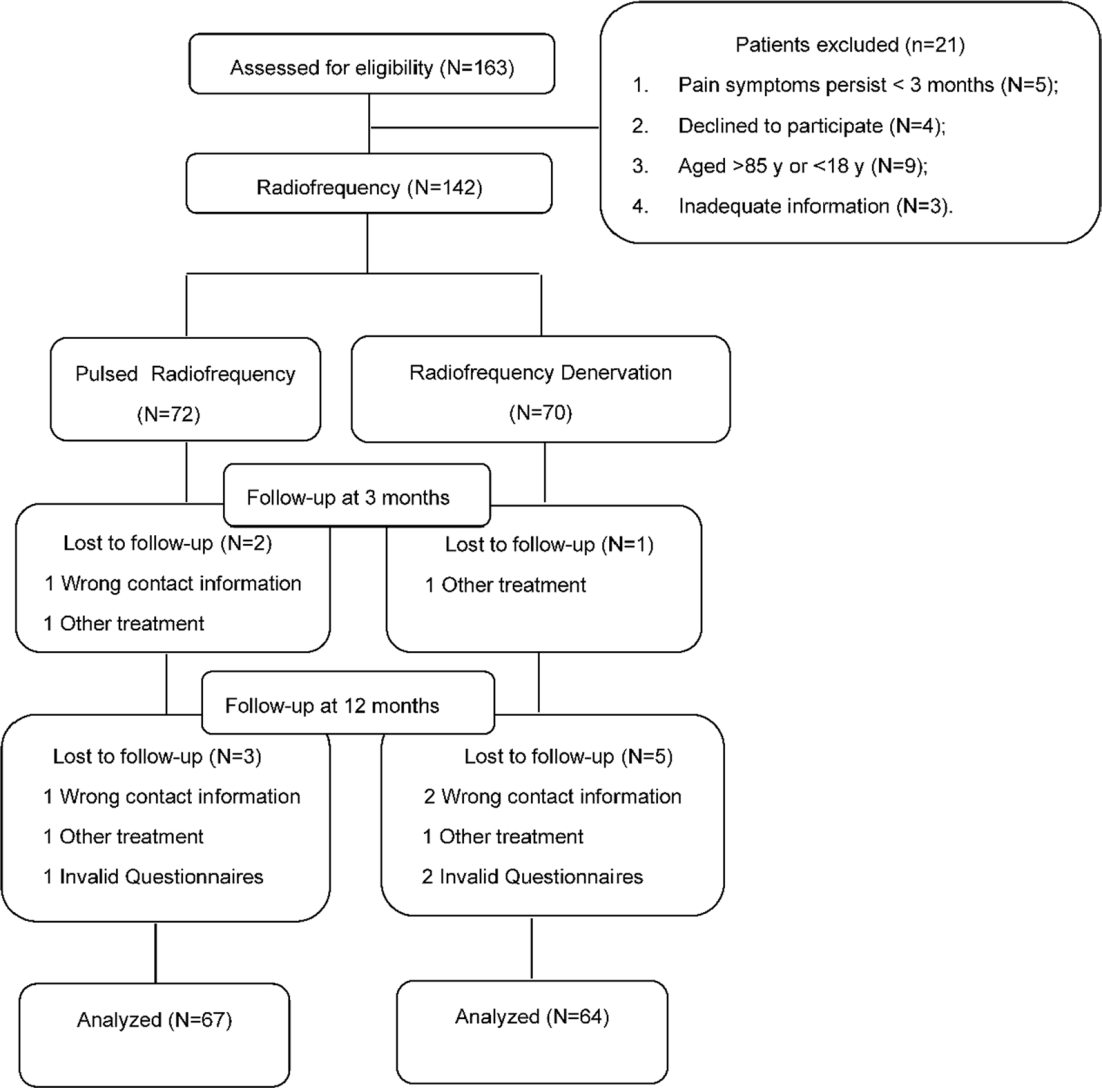
Flow of patients through enrollment in the two groups

Twenty-one patients were excluded from the study because of pain symptoms lasting < 3 months (*n* = 5), refusal to participate (*n* = 4), age > 85 or < 18 years (*n* = 9) and insufficient information (*n* = 3). A total of 142 patients were included in the study (Fig. 1).

### Measurements

All the selected patients were divided into the PRF group and the RD group. Pain assessment and quality of life were measured using a visual analog scale (VAS) and Short-Form36 (SF-36) questionnaire, respectively. Pain was assessed using the VAS, which is a reliable measure of subjective phenomena of various qualities of pain. The SF-36 questionnaire contains eight items including physical functioning (PF), role limitation physical (RP), bodily pain (BP), general health (GH), role of emotion (RE), vitality (VT) and mental health (MH). SF-36 has previously been validated for patients with chronic non-malignant pain. The Roland-Morris questionnaire (RMQ) and the Oswestry disability index questionnaire (ODI) were used to assess lumbar function.​ Patients were evaluated using the SF-36 questionnaire, VAS, RMQ and ODI, and administered either as clinician-administered questionnaire or as a mailed questionnaire, before therapy (baseline), and at 3 and 12 months after therapy.

### Technique

**Fig. 2:**
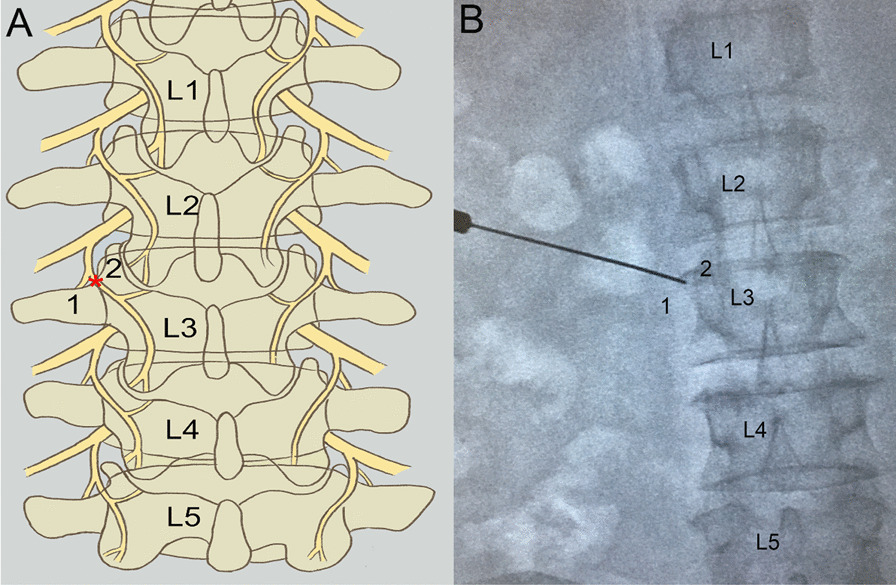
Anatomy of the posterior branch of the lumbar spinal nerve and X-ray guided radiofrequency needle insertion at target position. **A**: Branching of the posterior ramus of the lumbar spinal nerve at L3 (1: transverse process, 2: superior articular process, *: target position). **B**: Radiofrequency for the posterior ramus of the spinal nerve at L3. (Anterior–posterior fluoroscopy, 1: transverse process, 2: superior articular process)

Although there are numerous causes of lumbar facet joint pain, compression of the posterior branches of the lumbar spinal nerves is viewed as a significant factor. Lumbar facet joint pain can be treated with radiofrequency treatment. All patients required a local nerve block to diagnose lumbar joint pain before the therapy. The patient was placed in a prone position. 0.5 ml of 2% lidocaine was injected to the posterior branch of the lumbar spinal nerve. Patients with at least a 50% reduction in VAS score measured 30 min after injection were eligible for the next step. Under the guidance of X-rays, a 22-gauge needle was advanced into the origin of the transverse and the superior articular process. On the X-ray orthopantomograph, the needle tip is situated at the junction of the outer edge of the superior articular eminence and the superior border of the transverse eminence (Fig. 2). Sensory stimulation (50 Hz) and motor stimulation (2 Hz) were conducted to ensure the needle at the posterior branches of the lumbar spinal nerves. Patients could experience pain in the same location they did before the procedure. In the meantime, the process of PRF treatment was as follows: the tip temperature being at 42 °C, the voltage at 45 V, the frequency 2 Hz for 120 s, three cycles, and the parameters of RD treatment were: the tip temperature being at 80 °C, the voltage at 45 V, the frequency 2 Hz for 120 s, three cycles.

### Statistics

SPSS software, version 22.0 (IBM-SPSS, Armonk, NY, USA) and GraphPad Prism software (GraphPad Software, Inc., La Jolla, CA, USA) were used for statistical analysis. Quantitative data were expressed as mean ± standard deviation. Continuous data were compared by using an independent Student’s *t* test. A Chi-squared test or Fischer exact test was used to analyze categorical variables. Quality-of-life scores were assessed using the Mann–Whitney U test. A *p* value of < 0.05 was considered statistically significant.

## Results

A total number of 163 subjects with lumbar facet joint pain were selected for the study. Twenty-one patients were excluded from the study for various reasons (Fig. 1). One hundred and forty-two patients were assigned into the PRF group (*N* = 72) and the RD group (*N* = 70). In both groups (PRF and RD groups), the vast majority of patients were female and had bilateral low back pain. Patients in the PRF group were slightly older on average. There were no significant differences in baseline characteristics between the two groups (Table 1).

**Table 1:** Demographics and clinical characteristic of patients with lumbar facet joint pain in the two groups

Variable	PRF (*n* = 72)	RD (*n* = 70)	*p*
Age, Mean ± SD, range	63.2 ± 11.3 (30–81)	61.0 ± 13.0 (22–83)	0.291
Male/female	23/49	15/55	0.157
Smoking, (%)	18 (25.0)	14 (20.0)	0.476
Alcohol intakes, (%)	31 (43.1)	29 (41.4)	0.844
Side of symptoms, n (%)			0.320
Right or left, (%)	25 (34.7)	30 (42.9)	
Both, (%)	47 (65.3)	40 (47.1)	
Onset pain, n (%)			0.723
Suddenly, (%)	14 (19.4)	12 (17.1)	
Gradually, (%)	58 (80.6)	58 (82.9)	

### Pain assessment

**Fig. 3:**
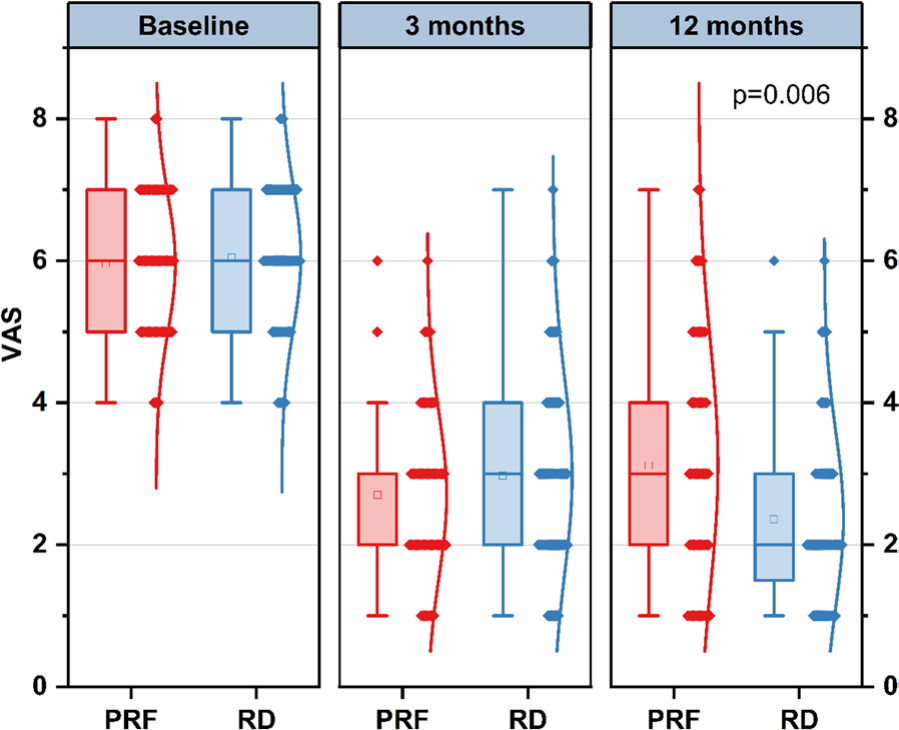
Results of the pain score of patients with lumbar facet joint pain before therapy and at 3, 12-month follow-up

There was no statistically significant difference concerning the pain score between the two groups before the therapy and at a 3-month follow-up. However, a significant reduction in pain score from a mean of 6.04 before therapy to 2.37 after 12 months of follow-up was found in the RD group (*p* < 0.001). Remarkable differences in pain control were observed in both groups of patients at 12 months (3.09 ± 1.72 vs. 2.37 ± 1.22, *p* = 0.006) (Fig. 3).

### Lumbar function assessment

**Fig. 4:**
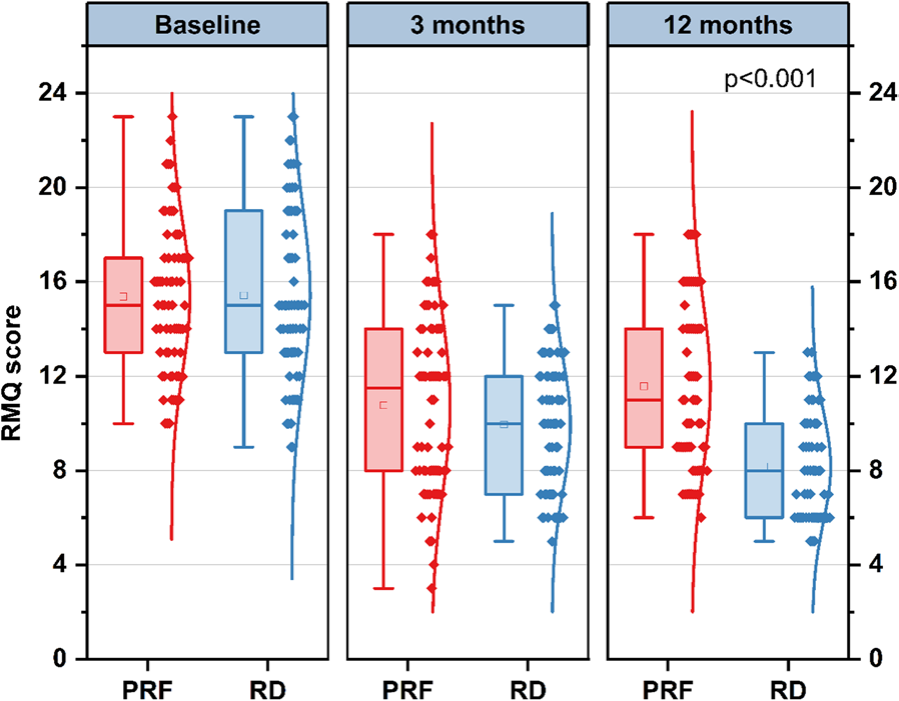
Results of RMQ score at different time points

Before therapy, there were no significant differences concerning the RMQ score between the two groups (15.36 ± 3.16 vs. 15.41 ± 3.69, *p* = 0.927). Although the RMQ score was high in the PRF group at 3 months after therapy, there was no statistically significant difference between the groups (10.77 ± 3.67 vs. 9.96 ± 2.75, *p* = 0.1.41). Compared with the PRF group, a remarkable reduction in the RMQ score was observed in the RD group of patients, as assessed 12 months after therapy (11.58 ± 3.58 vs.. 8.17 ± 2.34, *p* < 0.001) (Fig. 4).

**Fig. 5:**
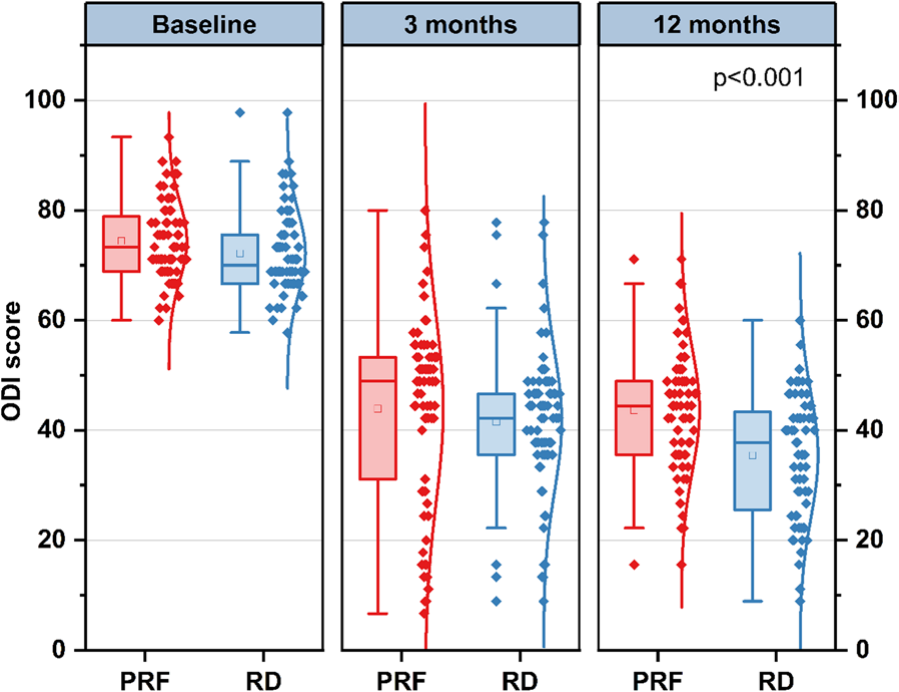
Results of ODI score at different time points

Regarding the ODI score, similar results were observed between the PRF and RD groups before therapy and at 3-month follow-up (74.44 ± 7.18 vs. 72.19 ± 7.55, *p* = 0.070; 43.90 ± 17.05 vs. 41.59 ± 12.59, *p* = 0.362). A significant reduction in ODI score was observed between the two groups at 12-month follow-up (43.65 ± 11.01 vs. 35,42 ± 11.32, *p* < 0.001) (Fig. 5).

### Quality-of-life assessment

Before therapy, patients with lumbar facet joint pain had poor quality of life. No significant difference was detected between the two groups (*p* > 0.05). At 3-month follow-up, 139 patients were included in this study (PRF group, *n* = 70; RD group, *n* = 69). All eight domains of the SF-36 score were almost identical in both groups (*p* > 0.05). Similar results were observed in the total SF-36 score between the two groups (51.99 ± 8.44 vs. 51.64 ± 7.32, *p* = 0.544). However, at 12-month follow-up, the eight domains of the SF-36 and the total score were significantly higher in the RD group compared to the PRF group (*p* < 0.05) (Table 2).

**Table 2:** Health-related quality-of-life outcomes at 3 months, 12 months after therapy

SF-36 domains	Follow-up at 3 months		p*	Follow-up at 12 months		p*
	PRF (*n* = 70)	RD (*n* = 69)		PRF (*n* = 67)	RD (*n* = 64)	
PF	60.79 ± 14.93	60.71 ± 16.05	0.970	57.09 ± 20.25	68.28 ± 13.22	0.001
RP	43.21 ± 23.67	40.17 ± 26.30	0.600	47.01 ± 22.83	58.20 ± 24.01	0.004
BP	43.71 ± 17.63	46.43 ± 13.30	0.157	64.48 ± 16.72	73.75 ± 16.48	0.002
GH	46.79 ± 29.42	42.57 ± 23.36	0.090	53.51 ± 19.29	62.89 ± 20.06	0.044
V	60.71 ± 22.48	55.79 ± 28.83	0.107	59.25 ± 13.29	66.33 ± 17.46	0.038
SF	58.94 ± 18.41	60.64 ± 16.71	0.623	61.46 ± 18.71	79.72 ± 13.75	< 0.001
RE	36.14 ± 30.52	43.80 ± 27.69	0.094	48.28 ± 25.01	59.94 ± 23.33	0.017
MH	65.60 ± 15.25	62.46 ± 16.25	0.059	76.48 ± 20.31	85.75 ± 11.97	0.024
Total score	51.99 ± 8.44	51.64 ± 7.32	0.544	58.45 ± 6.97	69.36 ± 6.43	< 0.001

Values are expressed as means ± SDs

*PF*, Physical function; *RP*, Role physical; *BP*, Bodily pain; *GH*, General health; *V*, Vitality; *SF*, Social function; *RE*, Role of emotion; *MH*, Mental health

*Mann–Whitney U test

### Complications

There were no serious adverse events over 12 months in either group. We found that six (4.2%) patients in the RD group had adverse events within 1 month. Skin numbness occurred in three patients. Mild pain such as burning and pinking at the puncture site in two patients. One patient experienced a decrease in back muscle strength and back muscle fatigue. These complications disappeared in 3 weeks without any treatment. There were no serious adverse events in the PRF group.

## Discussion

Lumbar facet joint pain is a disease that can significantly and globally impair all aspects of quality of life. Patients with lumbar facet joint pain experience moderate-to-severe pain intensity. Due to the prevalence of lumbar facet joint pain, pain control and improvement in quality of life are considered the most important outcome measures when evaluating therapeutic options.

Until recently, the treatment of lumbar facet joint pain has been a challenge. Many techniques such as nonsteroidal anti-inflammatory drugs, lifestyle modifications, nerve blocks and steroid injections have been used to treat lumbar facet joint pain, but these treatments have been controversial. Radiofrequency is an effective option for lumbar facet joint pain and should be considered for patients who have not responded to conservative medical therapy. Chang et al. [19] have found that intra-articular PRF was relatively simple to perform and produced good, long-lasting effects without serious complications. A meta-analysis of randomized controlled trials found strong evidence for the use of radiofrequency denervation to reduce pain in low back pain [20]. However, some studies reported that PRF was easy to relapse and has short-term pain relief [17, 18]. Juch et al. [21] found that RD did not improve chronic low back pain. In our study, we found that both PRF and RD achieved pain relief. Compared with PRF, there was no significant difference in VAS in the RD group at short-term follow-up (3 months). However, in the long-term follow-up (12 months), the patients in the RD group had a lower pain score than those in the PRF group. This showed that RD was superior to PRF in terms of pain control.

Importantly, the ultimate goal of treatment for lumbar facet joint pain is to improve lumbar function and enhance the quality of life. It is well known that patients with lumbar facet joint pain have a substantially impaired quality of life. A previous study reported that radiofrequency denervation of the lumbar zygapophysial joint could result in a significant reduction in functional disability in patients with lumbar facet joint pain [22]. Similarly, Nath et al. [23] reported that there was a significant improvement in quality-of-life variables, global perception of improvement and generalized pain. Sansone et al. [12] reported that ODI scores decreased during the follow-up period in patients with PRF. In contrast, Leclaire et al. [17] reported that radiofrequency facet joint denervation may provide some short-term improvement in functional disability. However, there was no treatment effect on functional disability at 12 weeks. In the present study, we found that both treatment groups had an improvement in lumbar function and quality of life in the short-term outcomes (3-month follow-up). There was no statistical difference between the two groups. However, in the long-term outcome (12-month follow-up), RD significantly improved functional capacity and all sub-quality-of-life parameters in patients with lumbar facet joint pain.

As for the complications, only a few studies were designed to evaluate side effects. Duger et al. [24] and Lakemeier et al. [25] found no adverse effects of RD in patients with low back pain. While two studies reported that patients who underwent RD had mild lower limb weakness and non-painful paresthesias, these symptoms had disappeared at the follow-up [26, 27]. In fact, we found that six patients in the RD group had adverse events within 1 month. Skin numbness occurred in three patients. Mild pain such as burning and pinking at the puncture site in two patients. One patient experienced a decrease in back muscle strength and back muscle fatigue. These complications disappeared in 3 weeks without any treatment. No permanent complications were observed in both groups.

There is no gold standard for radiofrequency treatment in lumbar facet joint pain. The results of most studies have been inconsistent and may even be opposite [13–18]. Our study showed that PRF and RD had equivalent short-term outcomes in terms of pain control, lumbar function and quality of life. However, RD was superior to PRF because of better pain control, lower lumbar function score and higher SF-36 score in the long-term outcome. Possible factors were as follows. First, radiofrequency therapy has a different mechanism of pain relief. The PRF generator produces alternating repetitive electrical stimulation. The analgesic effects of PRF neuromodulation are induced by electromagnetic fields. PRF delivers a low-energy electric field in the form of rapid pulses to the target nerve tissue and associated microglia. This technique does not cause nerve damage or complications [28]. In contrast, RD generates high temperatures (70–90 °C) in neural tissue, which ablate the nerves or tissue [10]. The RD treatment is ablative rather than neuromodulation, which may be responsible for the better effect on lumbar facet joint pain. Second, the targets of radiofrequency therapy are different. Some studies have used the lumbar facet joint as a therapeutic target. Chang et al. [19] performed intra-articular PRF. Pain scores were significantly reduced at the follow-up. According to one review, radiofrequency treatment for the facet joint was currently considered the standard treatment of the lumbar facet joints pain [29]. However, most studies have focused on the posterior branches of lumbar spinal nerves. In the past, the lumbar posterior ramus of the spinal nerve was divided into the medial and lateral branches [30, 31], but more recent studies have moved beyond this viewpoint and have considered that the lumbar posterior ramus of the spinal nerve consists of three branches, the medial, lateral and intermediate branches [32, 33]. Anatomically, the medial branch originates from the stem of the lumbar posterior ramus on the superior side of the transverse process of the lower vertebra and reaches the area near the spinous process and the facet joints [34]. So far, there is no consensus on the target sites for radiofrequency therapy. Thirdly, the pain scoring system and the quality-of-life system were different. Lakemeier et al. [25] compared intra-articular lumbar facet joint steroid injections and lumbar facet joint RD in the treatment of low back pain. VAS, RMQ and ODI were used to evaluate the pan and the lumbar function. Juch et al. [21] used the numerical rating scale (NRS) to assess pain intensity. Functional status, health-related quality of life and general health were measured by the ODI, the 3-level EuroQol 5D Health Questionnaire and the RAND 36-item Health Survey, respectively. Kim et al. [6] studied 23 patients with low back pain undergoing PRF treatment. They used VAS and ODI to assess the results of treatment. Chang et al. [19] only used the NRS to assess the treatment effects. We found that most studies did not assess the quality of life. In our study, pain relief and lumbar function were measured using VAS, ODI and RMQ. In addition, quality of life was measured using the Short-Form36 (SF-36) questionnaire. Therefore, a standardized and uniform assessment system are very important, which can reduce the differences between similar studies.

This study has several limitations. First, a major difficulty was our inability to obtain accurate scores in patients with lumbar facet joint pain. Pain, lumbar function and quality of life were assessed by description questionnaires, which are subjective measures. In the study, all the questionnaires and follow-up data were recorded by one physician and all treatments were performed by the same doctor. Second, it was a single-center study and the sample size was small. Thirdly, we did not assess the recurrence rate after the radiofrequency.

In conclusion, radiofrequency is an effective and safe treatment option for patients with lumbar facet joint pain. RD could provide good and lasting pain relief, with significant improvement in lumbar function and quality of life at long-term follow-up.
